# Sete Pecados Mortais no Manejo da Hipertensão: Uma Análise Aprofundada dos Erros em uma Jornada de Riva-Rocci à Fibrose Miocárdica

**DOI:** 10.36660/abc.20240811

**Published:** 2025-04-16

**Authors:** Claudio Pinho, Heitor Moreno, José Francisco Kerr Saraiva, Fernanda Marciano Consolim-Colombo

**Affiliations:** 1 Pontifícia Universidade Católica de Campinas Campinas SP Brasil Pontifícia Universidade Católica de Campinas – Cardiologia, Campinas, SP – Brasil; 2 Clínica Pinho Valinhos SP Brasil Clínica Pinho – Cardiologia, Valinhos, SP – Brasil; 3 Universidade Estadual de Campinas Campinas SP Brasil Universidade Estadual de Campinas, Campinas, SP – Brasil; 4 Hospital das Clínicas Faculdade de Medicina Universidade de São Paulo São Paulo SP Brasil Instituto do Coração do Hospital das Clínicas da Faculdade de Medicina da Universidade de São Paulo, São Paulo, SP – Brasil

**Keywords:** Hipertensão, Viés, Ciência

## Abstract

A medicina é talvez a única ciência que valoriza mais o conhecimento das publicações científicas mais recentes do que sua história ao longo do tempo. A epistemologia médica mostra que alguns erros e acertos estão tão próximos que muitas vezes não os diferenciamos prontamente. A produção do conhecimento médico nos faz entender que o conhecimento é transitório e as teorias precisam ser revalidadas, retificadas ou lapidadas, quando não destruídas e reconstruídas sobre outras bases; paradigmas que se renovam movimentam a ciência. Com essa visão crítica, tornou-se necessário acessar quanto conhecimento sobre hipertensão arterial foi construído ao longo dos últimos 130 anos, desde que a aferição da pressão arterial começou a ser difundida e se tornou rotina na prática médica até os dias atuais. A presente revisão crítica focou em erros na interpretação do conhecimento adquirido, sete dos quais foram identificados, profundamente discutidos e condenados como pecados devido à demora em serem reconhecidos, permitindo assim que a vida das pessoas com esta patologia cardiovascular fosse encurtada.

## Graphical abstract

**Figura Central:**
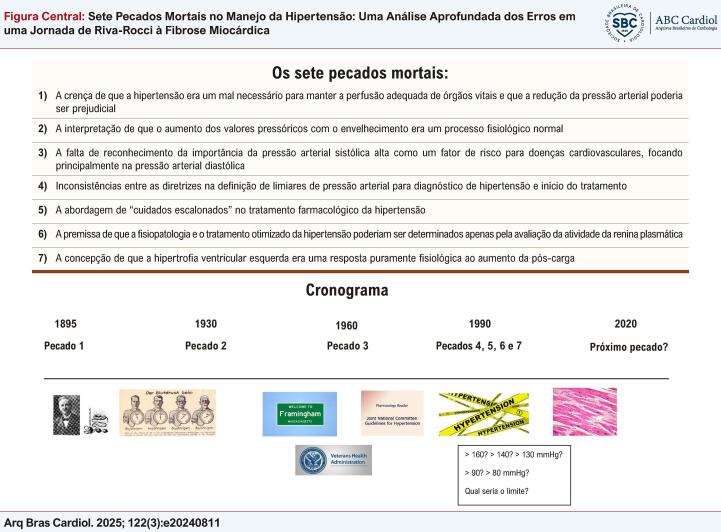
Sete Pecados Mortais no Manejo da Hipertensão: Uma Análise Aprofundada dos Erros em uma Jornada de Riva-Rocci à Fibrose Miocárdica

## Introdução

“O objetivo da Ciência não é abrir a porta para a sabedoria sem fim, mas estabelecer um limite para o erro sem fim” (de *Leben des Galilei*, 1938). Essa citação instigante de Bertolt Brecht prepara o cenário para o texto de Marvin Moser de 1997 sobre a “Evolução do Tratamento da Hipertensão da década de 1940 até o JNC V”, no qual ele afirma que “Existem poucas histórias na história da Medicina que são repletas de mais erros ou equívocos do que a história da hipertensão e seu tratamento”.^1^ Uma década depois, Moser revisitou o assunto em “Perspectivas Históricas sobre o Manejo da Hipertensão”,^2^ abordando o período entre 1950 e 2006. Na ciência, os erros geralmente precedem os acertos, oferecendo lições valiosas para avanços futuros.

Os erros têm sido parte integrante de nossas vidas desde a infância. O “jogo dos sete erros”, no qual comparamos meticulosamente duas imagens para identificar sete discrepâncias, chamadas de “erros”, cativava as nossas mentes jovens. Inspirando-nos nesse conceito, apresentamos uma revisão abrangente que explora sete equívocos significativos encontrados ao longo dos 130 anos de evolução do conhecimento em torno da hipertensão arterial (Figura Central). Nosso objetivo é atualizar esses “erros” para alinhá-los ao estado atual do entendimento científico.

A presente revisão aprofundada, motivada pela importância crítica do assunto, expande a citação de Bertolt Brecht, sugerindo que o objetivo da ciência não é apenas limitar erros infinitos, mas também evitar que eles se tornem pecados mortais. A escolha de enquadrar erros como pecados decorre da percepção de que atrasos na aquisição de conhecimento levaram a inúmeras perdas de vidas evitáveis devido à patologia em questão.

Apesar dos avanços significativos em nossa compreensão da hipertensão, várias questões permanecem sem resposta, por exemplo: as metas ideais de pressão arterial para diferentes populações de pacientes,^3^ o papel de novos biomarcadores e técnicas de imagem na detecção precoce e no tratamento de danos a órgãos relacionados à hipertensão,^4^ e o impacto dos determinantes sociais da saúde e das disparidades de saúde nos desfechos da hipertensão.^5^

À medida que navegamos pelas complexidades da pesquisa sobre hipertensão, é crucial reconhecer as lições aprendidas com equívocos do passado, ao mesmo tempo em que permanecemos abertos a novas descobertas que poderão desafiar nossa compreensão atual. Ao fazer isso, podemos continuar a refinar nossas abordagens para prevenção, diagnóstico e tratamento, melhorando os desfechos para pacientes com essa condição prevalente e potencialmente mortal.

### Os sete pecados mortais

1) A crença de que a hipertensão era um mal necessário para manter a perfusão adequada de órgãos vitais e que a redução da pressão arterial poderia ser prejudicial

O primeiro pecado, em nossa opinião, ocorreu por volta de 1895, quando Scipione Riva-Rocci publicou seu “novo esfigmomanômetro” na *Gazzetta Medica di Torino*. Na mesma época, a vasculopatia da hipertensão arterial foi descrita por Clifford Albutt com o termo alemão “*Essentielle Hypertonie*”, traduzido para o inglês como “*essential hypertension*” (hipertensão essencial), carregando consigo o conceito de que altos níveis de pressão arterial eram essenciais para superar a resistência das arteríolas comprometidas e perfundir os tecidos.^6,7^ Portanto, diminuir a pressão teria, supostamente, o efeito de piorar a perfusão tecidual e, consequentemente, não seria prudente fazê-lo. Embora Nikolai Sergeyevich Korotkov tenha descrito em 1905 que a ausculta acoplada a um esfigmomanômetro poderia acrescentar informações sobre os níveis diastólicos e a disseminação rotineira na prática clínica de medição da pressão arterial, sua redução era aconselhada apenas em casos de hipertensão maligna.

O conceito de níveis pressóricos elevados como um processo de defesa para evitar a diminuição do fluxo sanguíneo tecidual permaneceu arraigado até a década de 1960.^8,9^ Prova disso é a publicação em 1955 de George A. Perera, que descreveu as complicações de 500 pacientes hipertensos não tratados e sua sobrevida média em anos após o acometimento de lesões de órgãos-alvo aos níveis cardíaco (4 a 8 anos), renal (1 a 5 anos) e cerebral (1 a 4 anos).^10,11^ Na época, o entendimento era de que a hipertensão arterial, que reduziria a sobrevida em cerca de 15 a 20 anos em comparação aos pacientes normotensos, tinha uma fase descomplicada e assintomática, em que a orientação educacional sobre a patologia era a única recomendação, sem intervenção terapêutica, e uma fase complicada e sintomática em que a tentativa de redução da pressão arterial deveria ser feita com muito cuidado.^11,12^

No final da década de 1960, começou a se tornar robusto o conceito de que níveis pressóricos elevados eram responsáveis pela agressão e não um mecanismo de defesa contra a perfusão comprometida. Os responsáveis por essa mudança de paradigma foram inicialmente dados obtidos do Framingham Heart Study, cujo acompanhamento prospectivo havia começado em 1948,^13^ e do Veterans Administration Study Group.^14,15^ A questão mudou de “Quanto de acometimento da Hipertensão Arterial justifica seu tratamento?” para “Quão cedo se deve iniciar o tratamento para evitar ou reduzir significativamente a ocorrência de danos irreversíveis?”.^16^ As evidências começaram a mostrar definitivamente que quanto maior o nível de pressão arterial, maior o risco, independente de outras variáveis, de ser acometido por complicações cardiovasculares como insuficiência cardíaca, eventos coronários, acidente vascular cerebral e danos às funções renais. Se associado a comorbidades como diabetes, aterosclerose, tabagismo e obesidade, esse risco seria aumentado. Com essas informações e ainda em memória à perda do presidente dos Estados Unidos Franklin D. Roosevelt devido a complicações cardíacas e neurológicas da hipertensão em 1945,^2,8,17^ foram propostas forças-tarefas para orientar o diagnóstico e tratamento de pacientes com essa relevante patologia.

Devemos enfatizar o papel fundamental que o Veterans Administration Cooperative Study (VACS) de 1967 e 1970 desempenhou no refinamento e redirecionamento de nossa compreensão e gestão da hipertensão.^14,15^ Esses ensaios clínicos emblemáticos forneceram a primeira evidência definitiva de que tratar pacientes com altos valores de pressão arterial diastólica (PAD) poderia reduzir significativamente a incidência de acidente vascular cerebral, insuficiência cardíaca congestiva e outras complicações cardiovasculares. Esses achados desafiaram a noção predominante de que a hipertensão era um mal necessário para manter a perfusão adequada dos órgãos e que reduzir a pressão arterial poderia ser prejudicial. Em vez disso, o VACS estabeleceu os benefícios do tratamento da hipertensão e forneceu os fundamentos para o desenvolvimento de diretrizes baseadas em evidências para o manejo da hipertensão. Os resultados desses ensaios também estimularam mais pesquisas sobre a fisiopatologia da hipertensão e o desenvolvimento de novos medicamentos anti-hipertensivos, preparando o cenário para o progresso notável que temos feito na redução do fardo das doenças cardiovasculares ao longo das últimas cinco décadas.

Com essa nova concepção, nasceu o Joint National Committee (JNC), o primeiro sendo lançado em 1977,^18^ e o segundo em 1980.^19^

### 2) A interpretação de que o aumento dos valores pressóricos com o envelhecimento era um processo fisiológico normal

**Figura 1:**
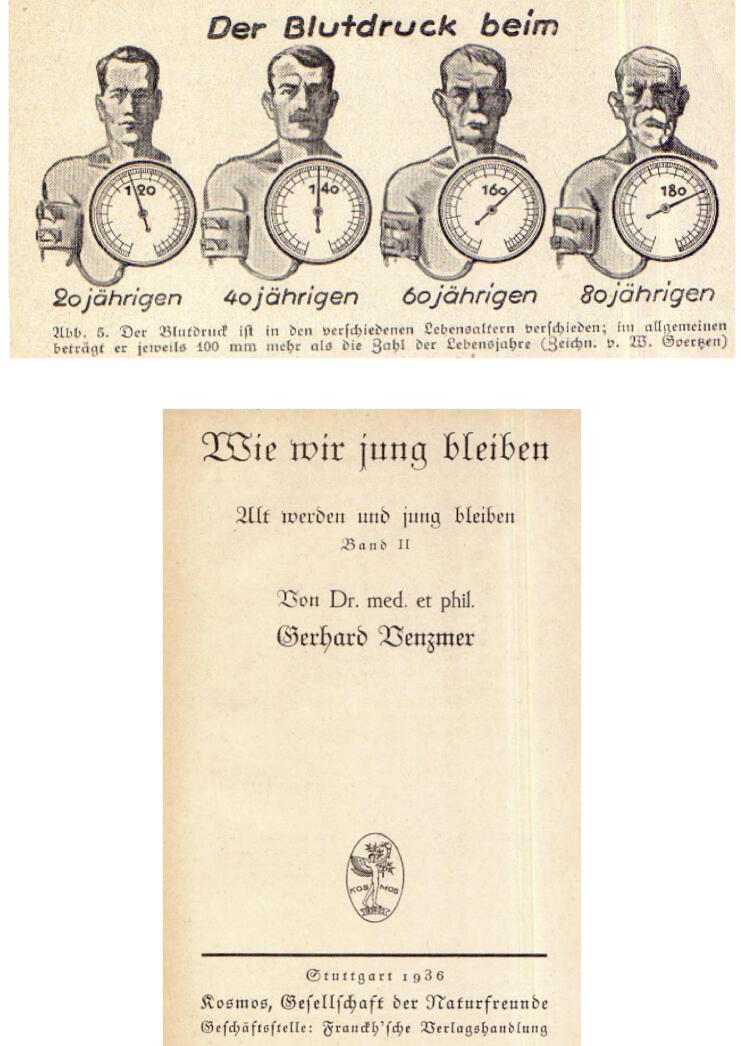
– Publicação alemã de 1936 diferenciando níveis “normais” em relação à idade.

Existe uma crença de longa data na comunidade médica de que a pressão arterial aumenta naturalmente com a idade e que esse aumento é benigno. A origem desse equívoco pode ser rastreada até estudos anteriores, como o relato de Fisher, de 1911, que sugeriu que uma pressão arterial sistólica (PAS) de 100 mmHg somada à idade do indivíduo era considerada normal.^20^ De fato, após a idade de 40 anos, era normal adicionar 10 mmHg para cada década da vida de uma pessoa. Assim, níveis de 160 mmHg eram considerados normais para a faixa etária de 60 anos, 170 mmHg para a faixa etária de 70 anos, e assim por diante (Figura 1). Esse conceito concordava com a ideia de que uma pressão arterial mais alta era necessária para manter a perfusão adequada para órgãos vitais em resposta a mudanças relacionadas à idade no sistema cardiovascular, como aumento da rigidez arterial e diminuição do débito cardíaco. No entanto, evidências mais recentes demonstraram claramente que o aumento da pressão arterial relacionado à idade não é uma necessidade fisiológica, mas sim um processo patológico que contribui para o aumento do risco cardiovascular.^21^

O Framingham Heart Study, que começou em 1948, foi um dos primeiros estudos a desafiar a noção de que o aumento da pressão arterial seria “normal” com o passar da idade. O estudo mostrou que indivíduos com pressão arterial mais alta tinham um risco maior de desenvolver doenças cardiovasculares, independentemente da idade.^22^ Estudos subsequentes mostraram consistentemente que o tratamento da hipertensão, mesmo em adultos mais velhos, pode reduzir significativamente o risco de eventos cardiovasculares e mortalidade. O Hypertension in the Very Elderly Trial (HYVET) demonstrou que o tratamento anti-hipertensivo em indivíduos com 80 anos ou mais reduziu o risco de acidente vascular cerebral, insuficiência cardíaca e mortalidade por todas as causas.^23^ Da mesma forma, o Systolic Blood Pressure Intervention Trial (SPRINT) mostrou que o controle intensivo da pressão arterial (visando PAS < 120 mmHg) reduziu o risco de eventos cardiovasculares e mortalidade por todas as causas em adultos com 50 anos ou mais, incluindo aqueles com 75 anos ou mais.^24^

As diretrizes atuais para o tratamento da hipertensão arterial enfatizam a importância do tratamento da hipertensão com base no perfil de risco cardiovascular de um indivíduo, em vez de limites específicos da idade.^25,26^ As diretrizes recomendam uma pressão arterial alvo de < 140/90 mmHg para a maioria dos adultos, com uma meta menor de < 130/80 mmHg para aqueles com alto risco cardiovascular, como indivíduos com diabetes, doença renal crônica ou doença cardiovascular estabelecida. Além disso, evidências atuais demonstram claramente que tratar a hipertensão, mesmo em pacientes idosos, pode reduzir significativamente a morbimortalidade cardiovascular. Profissionais de saúde devem se concentrar em avaliar e manejar a hipertensão com base no perfil geral de risco cardiovascular de cada indivíduo, em vez de confiar em limites arbitrários de pressão arterial específicos para cada idade.

### 3) A falta de reconhecimento da importância da pressão arterial sistólica alta como um fator de risco para doenças cardiovasculares, focando principalmente na pressão arterial diastólica

Nos dois primeiros documentos do JNC (1977 e 1980),^18,19^ o diagnóstico e a orientação terapêutica foram baseados apenas nos níveis de PAD. Aqui, nos deparamos com o terceiro erro, que foi a desconsideração dos níveis de PAS para classificação, avaliação de risco e decisão terapêutica.

As seguintes consequências de não reconhecer a importância da PAS como um fator de risco para doenças cardiovasculares e focar principalmente na PAD podem ser significativas: a) Subestimação do risco cardiovascular: Ao enfatizar a PAD em vez da PAS, os médicos podem subestimar o risco cardiovascular geral dos pacientes, particularmente em indivíduos mais idosos, que são mais propensos a ter hipertensão sistólica isolada. Isso pode levar à estratificação de risco inadequada e ao manejo subótimo da hipertensão.^27^ b) Início tardio do tratamento: Se as decisões de tratamento forem baseadas principalmente na PAD, os pacientes com PAS elevada, mas PAD normal, podem não receber intervenções oportunas para reduzir sua pressão arterial. Esse atraso no início do tratamento pode permitir a progressão de danos cardiovasculares e aumentar o risco de resultados adversos.^28^ c) Controle inadequado da pressão arterial: Focar somente na PAD pode levar ao controle inadequado da pressão arterial em pacientes com PAS elevada. Isso é particularmente preocupante porque a PAS demonstrou ser um preditor mais forte de eventos cardiovasculares do que a PAD, especialmente em indivíduos mais idosos.^29^ d) Aumento da morbimortalidade cardiovascular: A falha em controlar adequadamente a PAS pode resultar em uma maior incidência de complicações cardiovasculares, como infarto do miocárdio, acidente vascular cerebral, insuficiência cardíaca e doença renal. Esse aumento da morbimortalidade coloca um fardo significativo sobre os pacientes, famílias e sistemas de saúde.^30^ e) Alocação inapropriada de recursos de saúde: Ao não identificar e tratar efetivamente os pacientes com PAS elevada, os recursos de saúde podem ser mal direcionados, levando ao aumento dos custos de saúde e à redução da eficácia geral dos programas de manejo da hipertensão.^31^ Reconhecer a importância da PAS como um fator chave de risco para doenças cardiovasculares é crucial para uma avaliação precisa do risco, um início oportuno do tratamento apropriado e um controle otimizado da pressão arterial. Essa compreensão levou a uma mudança de foco em direção à PAS nas diretrizes recentes sobre hipertensão, enfatizando a necessidade de uma abordagem abrangente ao manejo da pressão arterial para reduzir o risco cardiovascular e melhorar os desfechos dos pacientes.^3^

### 4) Inconsistências entre as diretrizes na definição de limiares de pressão arterial para diagnóstico de hipertensão e início do tratamento

Ao longo dos anos, após vários relatórios do JNC, diferentes diretrizes publicadas por várias sociedades científicas de hipertensão arterial, cardiologia e nefrologia, além de documentos dos Comitês de Especialistas da Organização Mundial da Saúde, notamos divergências nos valores de normalidade e alvos terapêuticos que foram adotados ao longo do tempo. Em nossa opinião, o quarto pecado no manejo da hipertensão é a inconsistência na definição de limiares de pressão arterial para diagnóstico e início do tratamento entre as diretrizes, o que resultou em confusão, cuidados subótimos e desfechos desfavoráveis. Esse problema é ainda mais agravado pela falta de atenção às intervenções não farmacológicas, como modificações no estilo de vida, durante esse período crucial.

As discrepâncias nos limiares de pressão arterial entre diferentes diretrizes, como as diretrizes ACC/AHA de 2017 (130/80 mmHg)^3^ em comparação com as diretrizes ESC/ESH de 2018 e as Diretrizes Brasileiras (140/90 mmHg),^25,26^ criaram inconsistência na prática clínica, potencialmente negligenciando a oportunidade de intervir com modificações no estilo de vida em pacientes com hipertensão limítrofe.^32^

O foco no tratamento farmacológico nas diretrizes pode inadvertidamente levar a uma ênfase exagerada na medicação, particularmente no que diz respeito a pacientes com hipertensão leve e baixo risco cardiovascular, que podem se beneficiar mais de mudanças no estilo de vida.^33^ Brunström e Carlberg (2018)^34^ sugeriram que o tratamento anti-hipertensivo nessa população pode não fornecer benefícios significativos e pode até causar danos, destacando a necessidade de uma abordagem mais equilibrada que priorize mudanças no estilo de vida.

É compreensível e saudável que as sociedades científicas possam divergir em relação a valores ou limites considerados aceitáveis para o tratamento de várias doenças. Porém, de uma perspectiva de saúde populacional, seria oportuno e extremamente útil para as equipes de saúde se sociedades especializadas lideradas por entidades científicas globais pudessem se alinhar com recomendações convergentes.

Apesar dos benefícios bem estabelecidos das modificações de estilo de vida no manejo da hipertensão,^35^ sua incorporação na prática clínica permanece subótima.^36^ Mudanças no estilo de vida, como modificações da dieta, aumento da atividade física, controle do estresse e cessação do tabagismo, podem efetivamente reduzir a pressão arterial e melhorar a saúde cardiovascular.^37^ A falta de ênfase nessas intervenções nas diretrizes e na prática clínica pode levar a uma dependência excessiva da farmacoterapia e a desfechos subótimos para os pacientes. As diretrizes variam em suas recomendações para iniciar a medicação anti-hipertensiva, com algumas preconizando níveis pressóricos mais baixos em pacientes de alto risco e outras sugerindo uma abordagem mais conservadora. Há uma necessidade de maior clareza e consenso sobre quando e como iniciar intervenções no estilo de vida, pois elas podem ser eficazes na prevenção ou no atraso da necessidade de farmacoterapia.^38^

Concluindo, abordar as inconsistências na definição de limiares de pressão arterial para diagnóstico de hipertensão e início do tratamento em todas as diretrizes, combinadas com a falta de atenção às intervenções não farmacológicas, requer um esforço concentrado para harmonizar as diretrizes, priorizar modificações no estilo de vida e fornecer orientação clara sobre quando e como iniciar intervenções farmacológicas e não farmacológicas. Ao fazer isso, podemos otimizar os cuidados da hipertensão e melhorar os desfechos dos pacientes.

### 5) A abordagem de “cuidados escalonados” no tratamento farmacológico da hipertensão

O quinto pecado é a abordagem de “cuidados escalonados” (“*stepped care*”) para o manejo da hipertensão, conforme preconizada pelas diretrizes iniciais do JNC, que envolvia iniciar o tratamento com um único agente anti-hipertensivo, normalmente um diurético tiazídico, e adicionar gradualmente outros medicamentos de forma escalonada se a pressão arterial permanecesse descontrolada.^39^ Essa abordagem presumia que a maioria dos pacientes poderia atingir o controle adequado da pressão arterial com um único agente e que adicionar vários medicamentos aumentaria o risco de efeitos colaterais.^40^ No entanto, essa estratégia única não levou em consideração as características individuais dos pacientes, como idade, comorbidades e fisiopatologia subjacente, que podem influenciar a escolha da terapia inicial e a necessidade de tratamento combinado.^41^

Estudos subsequentes mostraram que uma proporção significativa de pacientes, particularmente aqueles com hipertensão mais grave ou fatores de risco cardiovascular adicionais, podem exigir terapia combinada desde o início para atingir o controle otimizado da pressão arterial e reduzir o risco de complicações.^42^ O ensaio Hypertension Optimal Treatment (HOT), publicado em 1998, demonstrou que uma PAD alvo mais baixa (≤ 80 mmHg) alcançada por meio da terapia combinada foi associada a um risco reduzido de eventos cardiovasculares em pacientes de alto risco com hipertensão.^43^ O ensaio ACCOMPLISH, publicado em 2008, mostrou que uma combinação de um inibidor da enzima conversora de angiotensina (IECA) e um bloqueador dos canais de cálcio foi mais eficaz na redução de eventos cardiovasculares do que uma combinação de um IECA e um diurético tiazídico, destacando a importância de combinações específicas de medicamentos no manejo da hipertensão.^44^

A abordagem de tratamento escalonado também pode levar a atrasos no alcance das metas de pressão arterial e pode contribuir para uma adesão subótima ao tratamento devido à necessidade de ajustes frequentes de medicamentos. O ensaio STITCH, publicado em 2003, verificou que uma combinação de um único comprimido de um IECA e um bloqueador dos canais de cálcio resultou em melhor controle da pressão arterial e adesão em comparação a uma abordagem de tratamento escalonado usando os mesmos medicamentos separadamente.^45^

Essas limitações levaram a uma mudança em direção a uma abordagem mais personalizada para o tratamento da hipertensão, que enfatiza a seleção individualizada do tratamento com base nas características do paciente e o uso de terapia combinada quando necessário para atingir o controle oportuno e eficaz da pressão arterial. Diretrizes recentes recomendam iniciar o tratamento com uma combinação de dois medicamentos para a maioria dos pacientes com hipertensão, particularmente aqueles com PAS ≥ 150 mmHg ou PAD ≥ 90 mmHg.^3,25,26^

### 6) A premissa de que a fisiopatologia e o tratamento otimizado da hipertensão poderiam ser determinados apenas pela avaliação da atividade da renina plasmática

No início da década de 1970, o sistema renina-angiotensina-aldosterona (SRAA) foi reconhecido como um alvo crucial para o tratamento da hipertensão.^46^ Essa descoberta levou ao desenvolvimento de vários agentes farmacológicos que poderiam bloquear diferentes componentes do SRAA, como IECA, bloqueadores do receptor da angiotensina e antagonistas da aldosterona.^47^ Durante esse período, surgiu o conceito de Laragh ou LARAH (hipertensão essencial com renina baixa, renina normal ou renina alta), que orientou o tratamento de acordo com os níveis de renina.^48^ Esse conceito foi baseado na premissa de que a fisiopatologia e o tratamento otimizado da hipertensão poderiam ser determinados pela avaliação da atividade da renina plasmática (ARP).^48,49^ De acordo com o conceito LARAH, acreditava-se que os pacientes com hipertensão com renina baixa tinham um volume plasmático expandido que responderia melhor aos diuréticos, que reduzem o volume plasmático e aumentam os níveis de renina. Pacientes com hipertensão com renina normal foram considerados como tendo um equilíbrio entre vasoconstrição e volume plasmático que responderia a uma combinação de diuréticos e vasodilatadores. Em contraste, acreditava-se que pacientes com hipertensão com renina alta tinham vasoconstrição como mecanismo fisiopatológico primário, que se beneficiaria mais de medicamentos que bloqueiam o SRAA, como betabloqueadores ou IECA.^50^

Embora o conceito LARAH tenha fornecido um arcabouço para entender a heterogeneidade da hipertensão e orientar decisões de tratamento, sua utilidade clínica tem sido questionada ao longo do tempo. Várias limitações dessa abordagem foram identificadas. Primeiro, a acurácia e a reprodutibilidade das medições de ARP podem ser afetadas por vários fatores, como ingestão de sódio na dieta, posição corporal e hora do dia.^51^ Segundo, a relação entre a ARP e a pressão arterial nem sempre é direta, e há uma sobreposição considerável nos níveis de ARP entre diferentes subgrupos de pacientes com hipertensão.^52^ Terceiro, a resposta aos medicamentos anti-hipertensivos não é determinada apenas pelos níveis de ARP; outros fatores como idade, raça e comorbidades podem influenciar os desfechos do tratamento.^53^ Quarto, o conceito LARAH não leva em conta os múltiplos mecanismos envolvidos na regulação da pressão arterial e os benefícios potenciais da combinação de medicamentos com diferentes mecanismos de ação.^54^

Apesar dessas limitações, o conceito LARAH desempenhou um papel significativo no avanço de nossa compreensão do SRAA e seu envolvimento na hipertensão. Também abriu caminho para o desenvolvimento de terapias direcionadas que se tornaram uma pedra angular do gerenciamento moderno da hipertensão. Portanto, embora o conceito LARAH tenha fornecido um arcabouço valioso para entender o papel do SRAA na hipertensão e orientar decisões de tratamento com base nos níveis de renina, sua utilidade clínica foi limitada por vários fatores. No entanto, continua sendo um conceito histórico importante que contribuiu para a evolução do manejo da hipertensão e para o desenvolvimento de terapias direcionadas.

### 7) A concepção de que a hipertrofia ventricular esquerda era uma resposta puramente fisiológica ao aumento da pós-carga

O sétimo pecado na compreensão histórica da hipertensão foi o equívoco de que a hipertrofia ventricular esquerda (HVE) é uma resposta puramente fisiológica ao aumento da pós-carga causada pela pressão arterial elevada. Essa visão simplificada falhou em reconhecer os complexos mecanismos fisiopatológicos envolvidos no desenvolvimento da HVE e suas potenciais consequências prejudiciais.^55^

Nos primórdios da pesquisa sobre hipertensão, a HVE era considerada uma resposta adaptativa que ajudava o coração a lidar com o aumento da carga de trabalho imposta pela pressão alta. Essa visão era baseada na observação de que a HVE era um achado comum em pacientes com hipertensão e que parecia normalizar o estresse da parede e manter o débito cardíaco.^56^ Sua presença era inicialmente reconhecida pela sobrecarga ventricular esquerda vista no ECG ou pelas alterações na silhueta cardíaca na radiografia de tórax, com base em informações de correlações anatômicas-clínicas.^57,58^ Sabia-se que o envolvimento cardíaco da hipertensão era visualmente expresso macroscopicamente pelo aumento da espessura das paredes ventriculares esquerdas e sob microscopia pela hipertrofia dos cardiomiócitos. Com a introdução da ecocardiografia, foi possível medir essa espessura e aumentar a sensibilidade diagnóstica de danos a órgãos-alvo ao nível cardíaco em comparação ao ECG e à radiografia de tórax.^57^

Pesquisas subsequentes revelaram que a HVE não é meramente uma adaptação fisiológica, mas sim um processo complexo que envolve múltiplos mecanismos fisiopatológicos, incluindo ativação neuro-hormonal, inflamação e fatores metabólicos e genéticos.^59^ O tecido miocárdico é composto de miócitos, vasos, sistema de condução e arcabouço contendo fibroblastos e colágeno. No entanto, devemos lembrar que em danos a órgãos-alvo ao nível cardíaco, o aumento desses constituintes (miocárdio, colágeno e vasos) não é proporcional.^60-63^ Assim, a predominância do colágeno pode levar à disfunção diastólica devido a alterações no relaxamento ventricular esquerdo e neovascularização inadequada, o que pode comprometer a reserva da artéria coronária.^60-63^

O SRAA e o sistema nervoso simpático desempenham um papel crucial no desenvolvimento da HVE promovendo hipertrofia dos cardiomiócitos, fibrose intersticial e deposição de colágeno.^64^ Hoje sabemos que a HVE é causada por múltiplos gatilhos, sendo o SRAA autócrino e parácrino o mais importante. Portanto, o bloqueio farmacológico do SRAA é uma das principais ações que previnem danos a órgãos-alvo ao nível cardíaco. O SRAA pode manter outros gatilhos liberados ou mesmo potencializar vias de escape, levando à adaptação dos pacientes com hipertensão a um novo ambiente onde a matriz extracelular sofreria alterações que culminariam em fibrose intersticial miocárdica, a saber: estímulo à formação de colágeno tipo I e tipo III; aumento de glicoproteínas, glicosaminoglicanos e proteoglicanos, bem como aumento da produção de fatores de crescimento e proteases. O resultado seria predominantemente fibrose reativa e não reparadora.^57,59^ Além disso, a inflamação crônica de baixo grau, frequentemente presente em pacientes hipertensos, contribui para o desenvolvimento de HVE ao ativar vias pró-fibróticas e promover a remodelação da matriz extracelular.^65^ Resistência à insulina, obesidade e dislipidemia, que são comorbidades comuns em pacientes com hipertensão, podem exacerbar o desenvolvimento de HVE ao induzir estresse oxidativo e alterar o metabolismo do substrato miocárdico.^66^ De fato, já há evidências de uma associação entre fibrose intersticial miocárdica e fatores de risco para doença arterial coronária, como Lp(a), o que aumentaria o risco de desfechos isquêmicos nesses pacientes com hipertensão.^67^ Finalmente, polimorfismos genéticos em várias vias neuro-hormonais e de sinalização foram associados a uma maior suscetibilidade à HVE, sugerindo que o histórico genético individual pode modular a resposta hipertrófica à hipertensão.^68^

Excelentes revisões deste assunto têm sido publicadas recentemente.^60-63,69^

Além disso, a noção de que a HVE seja uma resposta puramente adaptativa foi desafiada por evidências que demonstram sua associação com desfechos cardiovasculares adversos, como insuficiência cardíaca, arritmias e morte cardíaca súbita.^70^ A HVE é agora reconhecida como um fator de risco independente para morbimortalidade cardiovascular, e sua regressão se tornou um alvo terapêutico no manejo da hipertensão.^71^ A visão inicialmente simplificada falhou em explicar os complexos mecanismos fisiopatológicos envolvidos no desenvolvimento da HVE e suas potenciais consequências prejudiciais. O reconhecimento da HVE como um processo mal adaptativo e um fator de risco independente para resultados cardiovasculares adversos levou a uma mudança de paradigma no tratamento da hipertensão, enfatizando a importância de prevenir e reverter a HVE para melhorar os desfechos dos pacientes.

É importante notar que, na história da medicina, durante séculos, vivemos no obscurantismo e muitos dos diagnósticos e tratamentos foram guiados pelo empirismo, tornando compreensível que erros tenham sido cometidos com bastante frequência nos últimos séculos.

No entanto, é justo registrar que, na era da medicina moderna, o refinamento esmagador da compreensão da fisiopatologia das doenças cardiovasculares e a validação de estratégias terapêuticas foram baseadas na difícil construção de conceitos essenciais que sustentaram os sucessos do nosso tempo.

Considerando esses sete pecados, vamos realizar uma análise aprofundada para determinar se ainda há questões importantes relacionadas a cada um deles que permanecem relevantes hoje. É crucial chamar a atenção para a possibilidade de que perguntas em aberto possam persistir em cada um desses assuntos.

**1) A crença de que a hipertensão era um mal necessário para manter a perfusão adequada de órgãos vitais e que a redução da pressão arterial poderia ser prejudicial**

Pergunta: Existem subpopulações específicas ou cenários clínicos em que a redução agressiva da pressão arterial pode ser prejudicial?

**2) A interpretação de que o aumento dos valores pressóricos com o envelhecimento era um processo fisiológico normal**

Pergunta: Quais são as metas ideais de pressão arterial para adultos mais velhos, considerando os riscos e benefícios potenciais do tratamento?

**3) A falta de reconhecimento da importância da pressão arterial sistólica alta como um fator de risco para doenças cardiovasculares, focando principalmente na pressão arterial diastólica**

Pergunta: Existe um fenômeno de curva J para PAS em que valores excessivamente baixos podem estar associados a um risco cardiovascular aumentado?

**4) Inconsistências entre as diretrizes na definição de limiares de pressão arterial para diagnóstico de hipertensão e início do tratamento**

Pergunta: Como podemos harmonizar as várias diretrizes para fornecer recomendações claras e consistentes sobre o manejo da hipertensão?

**5) A abordagem de “cuidados escalonados” no tratamento farmacológico da hipertensão**

Pergunta: Em quais situações uma abordagem de terapia combinada pode ser preferível à abordagem tradicional de tratamento escalonado para controle otimizado da pressão arterial e redução do risco cardiovascular?

**6) A premissa de que a fisiopatologia e o tratamento otimizado da hipertensão poderiam ser determinados apenas pela avaliação da atividade da renina plasmática**

Pergunta: Como podemos integrar novos biomarcadores e abordagens de medicina personalizada para melhor caracterizar fenótipos individuais de hipertensão e orientar a terapia direcionada?

**7) A concepção de que a hipertrofia ventricular esquerda era uma resposta puramente fisiológica ao aumento da pós-carga**

Pergunta: Quais são as estratégias mais eficazes para prevenir e reverter a hipertrofia ventricular esquerda patológica em pacientes com hipertensão, além do controle da pressão arterial de forma isolada?

Em resumo, é evidente que, apesar do progresso significativo feito na compreensão e tratamento da hipertensão, muitas perguntas importantes permanecem sem resposta. Essas questões não resolvidas abrangem vários aspectos do manejo da hipertensão, incluindo metas de pressão arterial, estratégias de tratamento e a fisiopatologia dos danos aos órgãos relacionados à hipertensão. Ao reconhecer essas lacunas de conhecimento e buscar ativamente respostas por meio de pesquisas e ensaios clínicos contínuos, podemos continuar a refinar nossa abordagem ao manejo da hipertensão e melhorar os desfechos para pacientes afetados por essa condição prevalente e potencialmente devastadora. É fundamental que permaneçamos vigilantes na identificação e no tratamento dessas questões persistentes para garantir o tratamento otimizado para indivíduos com hipertensão.
