# Supplementary material for: Sete Pecados Mortais no Manejo da Hipertensão: Uma Análise Aprofundada dos Erros em uma Jornada de Riva-Rocci à Fibrose Miocárdica
Source: Arq Bras Cardiol. 2025 Apr 16;122(3):e20240811. [Article in Portuguese] doi: 10.36660/abc.20240811 (PMC12129475; doi:10.36660/abc.20240811)
Supplement: Translation

# Translation: Seven Deadly Sins in Hypertension Management: In-Depth Analysis of the Errors on a Journey from Riva-Rocci to Myocardial Fibrosis

**DOI:** 10.36660/abc.20240811i

**Authors:** Claudio Pinho, Heitor Moreno, José Francisco Kerr Saraiva, Fernanda Marciano Consolim-Colombo

**Affiliations:** 1 Brazil Pontifícia Universidade Católica de Campinas – Cardiologia, Campinas, SP – Brazil; 2 Brazil Clínica Pinho – Cardiologia, Valinhos, SP – Brazil; 3 Brazil Universidade Estadual de Campinas, Campinas, SP – Brazil; 4 Brazil Instituto do Coração do Hospital das Clínicas da Faculdade de Medicina da Universidade de São Paulo, São Paulo, SP – Brazil

**Keywords:** Hypertension, Bias, Science

## Abstract

Medicine is perhaps the only science that values knowledge of the most recent scientific publications more than its history over time. Medical epistemology shows that some mistakes and successes are so close that we often do not readily differentiate between them. The production of medical knowledge makes us understand that knowledge is transitory and theories need to be revalidated, rectified, or polished, if not destroyed and built again on other bases; paradigms that are renewed move science. With this critical view, it was necessary to access how much knowledge about arterial hypertension has been built over the last 130 years, since the measurement of blood pressure began to be widespread and become routine in medical practice until the present day. This critical review was focused on errors in the interpretation of acquired knowledge, seven of which have been identified, deeply discussed, and condemned as sins due to the delay in being recognized, thus allowing the lives of people with this cardiovascular pathology to be shortened.

## Introduction

“The aim of Science is not to open a door to endless wisdom, but to put a limit on endless errors” (from *Leben des Galilei*, 1938). This thought-provoking quote by Bertolt Brecht sets the stage for Marvin Moser’s 1997 text on the “Evolution of Hypertension Treatment from the 1940s to JNC V,” in which he states that, “There are few stories in the history of Medicine that are filled with more errors or misconceptions than the story of hypertension and its treatment.”^1^ A decade later, Moser revisits the topic in “Historical Perspectives on the Management of Hypertension”,^2^ covering the period between 1950 and 2006. In science, mistakes often precede successes, offering valuable lessons for future advancements.

Errors have been an integral part of our lives since childhood. The game of “spot the difference,” in which we meticulously compared two images to identify seven discrepancies, playfully termed “errors,” captivated us as young minds. Drawing inspiration from this concept, we present a comprehensive review that explores seven significant misconceptions encountered throughout the 130-year evolution of knowledge surrounding arterial hypertension (Central Illustration). We aim to update these “errors” to align with the current state of scientific understanding.

This in-depth review, motivated by the critical importance of the topic, expands upon Bertolt Brecht’s quote, suggesting that the aim of science is not only to limit endless errors but also to prevent them from becoming deadly sins. The choice to frame errors as sins stems from the realization that delays in acquiring knowledge have led to countless preventable losses of life due to this pathology.

Despite significant advancements in our understanding of hypertension, several questions remain unanswered, for instance: the optimal blood pressure targets for different patient populations,^3^ the role of novel biomarkers and imaging techniques in the early detection and management of hypertension-related organ damage,^4^ and the impact of social determinants of health and health disparities on hypertension outcomes.^5^

As we navigate the complexities of hypertension research, it is crucial to acknowledge the lessons learned from past misconceptions while remaining open to new discoveries that may challenge our current understanding. By doing so, we can continue to refine our approaches to prevention, diagnosis, and treatment, ultimately improving outcomes for patients with this prevalent and potentially deadly condition.

## The seven deadly sins

### 1) The belief that hypertension was a necessary evil to maintain adequate perfusion of vital organs and that lowering blood pressure could be harmful

The first of them, in our opinion, occurred around 1895 when Scipione Riva-Rocci published his “new sphygmomanometer” in the *Gazzetta Medica di Torino*. At the same time, the vasculopathy of arterial hypertension was described by Clifford Albutt with the German term “*Essentielle Hypertonie*,” translated into English as “essential hypertension,” which carried with it the concept that high blood pressure levels were essential to overcome the resistance of compromised arterioles and perfuse the tissues.^6,7^ Therefore, lowering the pressure would have the effect of worsening tissue perfusion, and consequently, it would not be prudent to do so. Although Nikolai Sergeyevich Korotkov described in 1905 that auscultation coupled to a sphygmomanometer could add information on diastolic levels and routine dissemination in the clinical practice of measuring blood pressure, its reduction was only advised in cases of malignant hypertension.

The concept of high blood pressure levels as a defense process to prevent a decrease in tissue blood flow remained ingrained until the 1960s.^8,9^ Proof of this is the publication in 1955 by George A. Perera, which described the complications of 500 untreated patients with hypertension and their average survival in years after the involvement of target organ lesions at the cardiac level (4 to 8 years), renal (1 to 5 years), and cerebral (1 to 4 years).^10,11^ At the time, the understanding was that arterial hypertension, which would reduce survival by around 15 to 20 years compared to normotensive patients, had an uncomplicated and asymptomatic phase, where educational guidance on the pathology was the only recommendation without therapeutic intervention, and a complicated and symptomatic phase where the attempt to reduce blood pressure should be done with great care.^11,12^

At the end of the 1960s, the concept that high blood pressure levels were responsible for aggression and not a defense mechanism against compromised perfusion began to become robust. Those responsible for this paradigm shift were initially data obtained from the Framingham Heart Study, whose prospective follow-up had begun in 1948,^13^ and from the Veterans Administration Study Group.^14,15^ The question changed from “How much hypertensive disability justifies the treatment of hypertension?” to “How early must one start treatment in order to avoid or greatly reduce the occurrence of irreversible disabilities?”^16^ Evidence began to definitively show that the higher the blood pressure level, the greater the risk, independent of other variables, of being affected by cardiovascular complications such as heart failure, coronary events, strokes, and renal functional damage. If associated with comorbidities such as diabetes, atherosclerosis, smoking, and obesity, this risk would be increased. With this information and still in memory of the loss of United States President Franklin D. Roosevelt due to cardiac and neurological complications of hypertension in 1945,^2,8,17^ task forces were proposed to guide the diagnosis and treatment of patients with this relevant pathology.

We must emphasize the pivotal role that the Veterans Administration Cooperative Study (VACS) of 1967 and 1970 played in refining and redirecting our understanding and management of hypertension.^14,15^ These landmark clinical trials provided the first definitive evidence that treating patients with high diastolic blood pressure (DBP) values could significantly reduce the incidence of stroke, congestive heart failure, and other cardiovascular complications. These findings challenged the prevailing notion that hypertension was a necessary evil to maintain adequate organ perfusion and that lowering blood pressure could be harmful. Instead, the VACS established the benefits of treating hypertension and laid the foundation for the development of evidence-based guidelines for hypertension management. The results of these trials also spurred further research into the pathophysiology of hypertension and the development of new antihypertensive medications, setting the stage for the remarkable progress we have made in reducing the burden of cardiovascular disease over the past five decades.

With this new conception, the Joint National Committee (JNC) was born, the first being launched in 1977^18^ and the second in 1980.^19^

### 2) The interpretation that increase in blood pressure values with aging was a normal physiological process

There has been a long-standing belief in the medical community that blood pressure naturally increases with age and that this increase is benign. This misconception can be traced back to early studies, such as the 1911 report by Fisher, which suggested that a systolic blood pressure (SBP) of 100 mmHg plus the individual’s age was considered normal.^20^ Indeed, after the age of 40, it was normal to add 10 mmHg for each decade of a person’s life. Thus, levels of 160 mmHg were considered normal for the 60-year-old age group, 170 mmHg for the 70-year-old age group, and so on (Figure 1). This concept agreed with the idea that higher blood pressure was necessary to maintain adequate perfusion to vital organs in response to age-related changes in the cardiovascular system, such as increased arterial stiffness and decreased cardiac output. However, more recent evidence has clearly demonstrated that the age-related increase in blood pressure is not a physiological necessity, but rather a pathological process that contributes to increased cardiovascular risk.^21^

**Figure 1:** – German publication from 1936 differentiating “normal” levels in relation to age.

The Framingham Heart Study, which began in 1948, was one of the first to challenge the notion of “normal” blood pressure increasing with age. This study showed that individuals with higher blood pressure had a greater risk of developing cardiovascular disease, regardless of age.^22^ Subsequent studies have consistently shown that treating hypertension, even in older adults, can significantly reduce the risk of cardiovascular events and mortality. The Hypertension in the Very Elderly Trial (HYVET) demonstrated that antihypertensive treatment in individuals aged 80 years and older reduced the risk of stroke, heart failure, and all-cause mortality.^23^ Similarly, the Systolic Blood Pressure Intervention Trial (SPRINT) showed that intensive blood pressure control (targeting SBP < 120 mmHg) reduced the risk of cardiovascular events and all-cause mortality in adults aged 50 years and older, including those aged 75 and older.^24^

Current guidelines for the management of arterial hypertension emphasize the importance of treating hypertension based on an individual’s cardiovascular risk profile rather than age-specific thresholds.^25,26^ The guidelines recommend a target blood pressure of < 140/90 mmHg for most adults, with a lower target of < 130/80 mmHg for those at high cardiovascular risk, such as individuals with diabetes, chronic kidney disease, or established cardiovascular disease. Moreover, current evidence clearly demonstrates that treating hypertension, even in the elderly, can significantly reduce cardiovascular morbidity and mortality. Healthcare professionals should focus on assessing and managing hypertension based on an individual’s overall cardiovascular risk profile rather than relying on arbitrary age-specific blood pressure thresholds.

### 3) The lack of recognition of the importance of high systolic blood pressure as a risk factor for cardiovascular disease, focusing mainly on diastolic blood pressure

In the first two JNC documents (1977 and 1980),^18,19^ the diagnosis and therapeutic guidance were based only on DBP levels. Here, we come across the third error, which was the disregard for SBP levels for classification, risk assessment, and therapeutic decision.

The following consequences of failing to recognize the importance of SBP as a risk factor for cardiovascular disease and focusing mainly on DBP can be significant: a) Underestimation of cardiovascular risk: By emphasizing DBP over SBP, clinicians may underestimate the overall cardiovascular risk in patients, particularly in older individuals who are more likely to have isolated systolic hypertension. This can lead to inadequate risk stratification and suboptimal management of hypertension.^27^ b) Delayed initiation of treatment: If treatment decisions are based primarily on DBP, patients with elevated SBP but normal DBP may not receive timely interventions to lower their blood pressure. This delay in treatment initiation can allow the progression of cardiovascular damage and increase the risk of adverse outcomes.^28^ c) Inadequate blood pressure control: Focusing solely on DBP may lead to inadequate blood pressure control in patients with elevated SBP. This is particularly concerning because SBP has been shown to be a stronger predictor of cardiovascular events than DBP, especially in older individuals.^29^ d) Increased cardiovascular morbidity and mortality: Failure to adequately control SBP can result in a higher incidence of cardiovascular complications, such as myocardial infarction, stroke, heart failure, and kidney disease. This increased morbidity and mortality places a significant burden on patients, families, and healthcare systems.^30^ e) Misallocation of healthcare resources: By not effectively identifying and treating patients with elevated SBP, healthcare resources may be misdirected, leading to increased healthcare costs, and reduced overall effectiveness of hypertension management programs.^31^ Recognizing the importance of SBP as a key risk factor for cardiovascular disease is crucial for accurate risk assessment, timely initiation of appropriate treatment, and optimal blood pressure control. This understanding has led to a shift in focus towards SBP in recent hypertension guidelines, emphasizing the need for a comprehensive approach to blood pressure management to reduce cardiovascular risk and improve patient outcomes.^3^

### 4) Inconsistencies in defining blood pressure thresholds for hypertension diagnosis and treatment initiation across guidelines

Over the years, after several JNC reports, different guidelines published by several scientific societies of arterial hypertension, cardiology and nephrology, in addition to documents by World Health Organization Expert Committees, we noticed divergences in the normality values and therapeutic targets that have been adopted over time. In our opinion, the fourth sin in hypertension management is the inconsistency in defining blood pressure thresholds for diagnosis and treatment initiation across guidelines, which has led to confusion, suboptimal care, and poor outcomes. This issue is further compounded by the lack of attention to non-pharmacologic interventions, such as lifestyle modifications, during this crucial period.

The discrepancies in blood pressure thresholds across different guidelines, such as the 2017 ACC/AHA guidelines (130/80 mmHg)^3^ compared to 2018 ESC/ESH guidelines and Brazilian Guidelines (140/90 mmHg),^25,26^ have created inconsistency in clinical practice, potentially neglecting the opportunity to intervene with lifestyle modifications in patients with borderline hypertension.^32^

The focus on pharmacologic treatment in guidelines may inadvertently lead to an overemphasis on medication, particularly concerning for patients with mild hypertension and low cardiovascular risk, who may benefit more from lifestyle changes.^33^ Brunström and Carlberg (2018)^34^ suggested that antihypertensive treatment in this population may not provide significant benefits and may even cause harm, highlighting the need for a more balanced approach that prioritizes lifestyle changes.

It is understandable and healthy that scientific societies may differ regarding values or limits considered acceptable for the treatment of various illnesses. However, from a population health perspective, it would be timely and extremely useful for health teams if specialty societies led by global scientific entities could be aligned with converging recommendations.

Despite the well-established benefits of lifestyle modifications in hypertension management,^35^ their incorporation into clinical practice remains suboptimal.^36^ Lifestyle changes, such as dietary modifications, increased physical activity, stress management, and smoking cessation, can effectively lower blood pressure and improve cardiovascular health.^37^ The lack of emphasis on these interventions in guidelines and clinical practice may lead to an overreliance on pharmacotherapy and suboptimal patient outcomes. Guidelines vary in their recommendations for starting antihypertensive medication, with some advocating for lower blood pressure levels in high-risk patients and others suggesting a more conservative approach. There is a need for greater clarity and consensus on when and how to initiate lifestyle interventions, as they can be effective in preventing or delaying the need for pharmacotherapy.^38^

In conclusion, addressing the inconsistencies in defining blood pressure thresholds for hypertension diagnosis and treatment initiation across guidelines, combined with the lack of attention to non-pharmacologic interventions, requires a concerted effort to harmonize guidelines, prioritize lifestyle modifications, and provide clear guidance on when and how to initiate both pharmacologic and non-pharmacologic interventions. By doing so, we can optimize hypertension care and improve patient outcomes.

### 5) The “stepped care” approach in pharmacological treatment of hypertension

The fifth sin is the “stepped care” approach to hypertension management, as advocated by the early JNC guidelines, which involved initiating treatment with a single antihypertensive agent, typically a thiazide diuretic, and gradually adding other medications in a stepwise manner if blood pressure remained uncontrolled.^39^ This approach assumed that most patients could achieve adequate blood pressure control with a single agent and that adding multiple drugs would increase the risk of side effects.^40^ However, this one-size-fits-all strategy failed to consider individual patient characteristics, such as age, comorbidities, and underlying pathophysiology, which may influence the choice of initial therapy and the need for combination treatment.^41^

Subsequent studies have shown that a significant proportion of patients, particularly those with more severe hypertension or additional cardiovascular risk factors, may require combination therapy from the outset to achieve optimal blood pressure control and reduce the risk of complications.^42^ The Hypertension Optimal Treatment (HOT) trial, published in 1998, demonstrated that a lower target DBP (≤ 80 mmHg) achieved through combination therapy was associated with a reduced risk of cardiovascular events in high-risk patients with hypertension.^43^ The ACCOMPLISH trial, published in 2008, showed that a combination of an angiotensin-converting enzyme inhibitor (ACEI) and a calcium channel blocker was more effective in reducing cardiovascular events than a combination of an ACEI and a thiazide diuretic, highlighting the importance of specific drug combinations in hypertension management.^44^

The stepped care approach may also lead to delays in achieving blood pressure goals and may contribute to suboptimal adherence to treatment due to the need for frequent medication adjustments. The STITCH trial, published in 2003, found that a single-pill combination of an ACEI and a calcium channel blocker resulted in better blood pressure control and adherence compared to a stepped care approach using the same medications separately.^45^

These limitations have led to a shift towards a more personalized approach to hypertension management, which emphasizes individualized treatment selection based on patient characteristics and the use of combination therapy when needed to achieve timely and effective blood pressure control. Recent guidelines recommend initiating treatment with a two-drug combination for most patients with hypertension, particularly those with SBP ≥ 150 mmHg or DBP ≥ 90 mmHg.^3,25,26^

### 6) The premise that the pathophysiology and optimal treatment of hypertension could be determined solely by assessing plasma renin activity

In the early 1970s, the renin-angiotensin-aldosterone system (RAAS) was recognized as a crucial target for the treatment of hypertension.^46^ This discovery led to the development of various pharmacological agents that could block different components of the RAAS, such as ACEIs, angiotensin receptor blockers, and aldosterone antagonists.^47^ During this period, the concept of Laragh or LARAH (low-renin, normal-renin, and high-renin essential hypertension) emerged, which guided treatment according to renin levels.^48^ This concept was based on the premise that the pathophysiology and optimal treatment of hypertension could be determined by assessing plasma renin activity (PRA).^48,49^ According to the LARAH concept, patients with low-renin hypertension were thought to have an expanded plasma volume that would respond best to diuretics, which reduce plasma volume and increase renin levels. Patients with normal-renin hypertension were considered to have a balance between vasoconstriction and plasma volume that would respond to a combination of diuretics and vasodilators. In contrast, patients with high-renin hypertension were believed to have vasoconstriction as the primary pathophysiological mechanism, which would benefit most from drugs that block the RAAS, such as beta blockers or ACEIs.^50^

While the LARAH concept provided a framework for understanding the heterogeneity of hypertension and guiding treatment decisions, its clinical utility has been questioned over time. Several limitations of this approach have been identified. First, the accuracy and reproducibility of PRA measurements can be affected by various factors, such as dietary sodium intake, body position, and time of day.^51^ Second, the relationship between PRA and blood pressure is not always straightforward, and there is considerable overlap in PRA levels among different subgroups of patients with hypertension.^52^ Third, the response to antihypertensive medications is not determined solely by PRA levels, and other factors such as age, race, and comorbidities can influence treatment outcomes.^53^ Fourth, the LARAH concept does not account for the multiple mechanisms involved in blood pressure regulation and the potential benefits of combining drugs with different mechanisms of action.^54^

Despite these limitations, the LARAH concept played a significant role in advancing our understanding of the RAAS and its involvement in hypertension. It also paved the way for the development of targeted therapies that have become a cornerstone of modern hypertension management. Therefore, while the LARAH concept provided a valuable framework for understanding the role of the RAAS in hypertension and guiding treatment decisions based on renin levels, its clinical utility has been limited by various factors. Nevertheless, it remains an important historical concept that has contributed to the evolution of hypertension management and the development of targeted therapies.

### 7) The conception that left ventricular hypertrophy was a purely physiological response to increased afterload

The seventh sin in the historical understanding of hypertension was the misconception that left ventricular hypertrophy (LVH) is a purely physiological response to the increased afterload caused by elevated blood pressure. This oversimplified view failed to recognize the complex pathophysiological mechanisms involved in the development of LVH and its potential detrimental consequences.^55^

In the early days of hypertension research, LVH was considered an adaptive response that helped the heart cope with the increased workload imposed by high blood pressure. This view was based on the observation that LVH was a common finding in patients with hypertension and that it seemed to normalize wall stress and maintain cardiac output.^56^ Its presence would initially be seen by the left ventricular overload seen on the ECG or changes in the cardiac silhouette on the chest X-ray, based on information from anatomical-clinical correlations.^57,58^ It was known that the cardiac involvement of hypertension was visually expressed macroscopically by the increase in the thickness of the left ventricular walls and under microscopy by cardiomyocyte hypertrophy. With the introduction of echocardiography, it was possible to measure this thickness and increase the diagnostic sensitivity of cardiac target organ damage compared to ECG and chest radiography.^57^

Subsequent research revealed that LVH is not merely a physiological adaptation, but rather a complex process involving multiple pathophysiological mechanisms, including neurohormonal activation, inflammation, and metabolic and genetic factors.^59^ Myocardial tissue is composed of myocytes, vessels, conduction system, and scaffold containing fibroblasts and collagen. However, we must remember that, in cardiac target organ damage, the increase in these constituents (myocardium, collagen, and vessels) is not proportional.^60-63^ Thus, the predominance of the collagen could lead to diastolic dysfunction due to changes in left ventricular relaxation and inadequate neovascularization, which can compromise the coronary artery reserve.^60-63^

The RAAS and the sympathetic nervous system play a crucial role in the development of LVH by promoting cardiomyocyte hypertrophy, interstitial fibrosis, and collagen deposition.^64^ Today we know that LVH is caused by multiple triggers, with the autocrine and paracrine RAAS being the most important. Therefore, the pharmacological blockade of the RAAS is one of the main actions that prevent cardiac target organ damage. The RAAS may keep other triggers released or even enhance escape pathways, leading to the adaptation of patients with hypertension to a new environment where the extracellular matrix would undergo changes that would culminate in myocardial interstitial fibrosis, namely: stimulus to the formation of type I and type III collagen; an increase in glycoproteins, glycosaminoglycans, and proteoglycans; as well as an increase in the production of growth factors and proteases. The result would be predominantly reactive and non-reparative fibrosis.^57,59^ Moreover, chronic low-grade inflammation, often present in patients with hypertension, contributes to the development of LVH by activating pro-fibrotic pathways and promoting extracellular matrix remodeling.^65^ Insulin resistance, obesity, and dyslipidemia, which are common comorbidities in patients with hypertension, can exacerbate the development of LVH by inducing oxidative stress and altering myocardial substrate metabolism.^66^ Indeed, there is already evidence of an association between myocardial interstitial fibrosis and risk factors for coronary artery disease, such as Lp(a), which would increase the risk of ischemic outcomes in these patients with hypertension.^67^ Finally, genetic polymorphisms in various neurohormonal and signaling pathways have been associated with an increased susceptibility to LVH, suggesting that individual genetic background may modulate the hypertrophic response to hypertension.^68^

Excellent reviews of this topic have recently been published.^60-63,69^

Furthermore, the notion that LVH is a purely adaptive response has been challenged by evidence demonstrating its association with adverse cardiovascular outcomes, such as heart failure, arrhythmias, and sudden cardiac death.^70^ LVH is now recognized as an independent risk factor for cardiovascular morbidity and mortality, and its regression has become a therapeutic target in the management of hypertension.^71^ The initial oversimplified view failed to account for the complex pathophysiological mechanisms involved in the development of LVH and its potential detrimental consequences. The recognition of LVH as a maladaptive process and an independent risk factor for adverse cardiovascular outcomes has led to a paradigm shift in the management of hypertension, emphasizing the importance of preventing and reversing LVH to improve patient outcomes.

It should be noted that, in the history of medicine, for centuries, we lived in obscurantism and many of the diagnoses and treatments were guided by empiricism, making it understandable that mistakes were made quite frequently in recent centuries.

However, it is fair to record that, in the era of modern medicine, the overwhelming refinement of the understanding of the pathophysiology of cardiovascular diseases and the validation of therapeutic strategies have been based on the difficult construction of essential concepts that supported the successes of our time.

Considering these seven sins, let us perform an in-depth analysis to determine if there are still important questions related to each of them that remain relevant today. It is crucial to draw attention to the possibility that unresolved questions may persist within each of these topics.

**1) The belief that hypertension was a necessary evil to maintain adequate perfusion of vital organs and that lowering blood pressure could be harmful**

Question: Are there any specific subpopulations or clinical scenarios where aggressive blood pressure lowering may be detrimental?

**2) The interpretation that an increase in blood pressure values with aging was a normal physiological process**

Question: What are the optimal blood pressure targets for older adults, considering the potential risks and benefits of treatment?

**3) The lack of recognition of the importance of high systolic blood pressure as a risk factor for cardiovascular disease, focusing mainly on diastolic blood pressure**

Question: Is there a J-curve phenomenon for systolic blood pressure, where excessively low values may be associated with increased cardiovascular risk?

**4) Inconsistencies in defining blood pressure thresholds for hypertension diagnosis and treatment initiation across guidelines**

Question: How can we harmonize the various guidelines to provide clear and consistent recommendations for hypertension management?

**5) The “stepped care” approach in pharmacological treatment of hypertension**

Question: In what situations might a combination therapy approach be preferable to the traditional stepped care approach for optimal blood pressure control and cardiovascular risk reduction?

**6) The premise that the pathophysiology and optimal treatment of hypertension could be determined solely by assessing plasma renin activity**

Question: How can we integrate novel biomarkers and personalized medicine approaches to better characterize individual hypertension phenotypes and guide targeted therapy?

**7) The conception that left ventricular hypertrophy was a purely physiological response to increased afterload**

Question: What are the most effective strategies for preventing and reversing pathological left ventricular hypertrophy in patients with hypertension, beyond blood pressure control alone?

In summary, it is evident that, despite the significant progress made in understanding and treating hypertension, many important questions remain unanswered. These unresolved issues span across various aspects of hypertension management, including blood pressure targets, treatment strategies, and the pathophysiology of hypertension-related organ damage. By acknowledging these knowledge gaps and actively seeking answers through ongoing research and clinical trials, we can continue to refine our approach to hypertension management and improve outcomes for patients affected by this prevalent and potentially devastating condition. It is imperative that we remain vigilant in identifying and addressing these persistent questions to ensure optimal care for individuals with hypertension.
